# Reliability and validation of the Japanese version of the cognitive distortion scale

**DOI:** 10.3389/fpsyg.2023.1261166

**Published:** 2024-06-11

**Authors:** Tomoya Takeda, Koudai Fukudome, Mina Nakano, Hidehiro Umehara, Kimiya Nakamura

**Affiliations:** ^1^Department of Psychological Sciences, University of Human Environments, Matsuyama, Japan; ^2^Department of Human and Social Studies, St. Catherine University, Matsuyama, Japan; ^3^Department of Psychology, Fukuyama University, Fukuyama, Japan; ^4^Department of Psychiatry, Graduate School of Biomedical Sciences, Tokushima University, Tokushima, Japan; ^5^Nakamura Clinic, Tokushima, Japan

**Keywords:** depression, cognitive distortion, CDS-J, cognitive theory, validation study

## Abstract

The cognitive distortion scale (CDS) is a self-rated measure to assess the degree of cognitive distortion which is 10 thinking errors commonly seen in depression. However, there is no scale to measure 10 types cognitive distortions specific to depression in Japan. Therefore, this study translated the CDS into Japanese (CDS-J), and examined its factor structure, validity, and reliability in a Japanese population. A total of 237 healthy individuals and 39 individuals with depression participated in this study. Confirmatory factor analysis indicated the appropriateness of the CDS-J’s 10-factor structure. Regarding convergent validity, CDS-J was significantly correlated with dysfunctional attitudes, negative automatic thoughts, and depression. Regarding discriminant validity, the CDS-J showed no significant correlation with positive automatic thoughts. The total CDS-J scores of the healthy participants and of those with major depression were compared. The results showed significant differences between groups. Finally, the CDS-J was found to have a high test–retest reliability. Therefore, the CDS-J is a valid and reliable tool for assessing cognitive distortions in Japan.

## 1. Introduction

Depression is a psychiatric disorder with core symptoms of consistent feelings of sadness and low motivation, and peripheral symptoms such as sleep disturbances, psychomotor agitation or inhibition, feelings of worthlessness and guilt, poor thinking and concentration, and suicidal ideation (American Psychiatric Association, 2013). The lifetime prevalence of depression varies regionally by economic status, ranging from 14.6% in high-income countries to 11.1% in middle- and low-income countries (Bromet et al., 2011), making it a common disorder that affects at least one in 10 people in their lifetime. Moreover, health losses associated with depression are significant. Among diseases affecting the disability-adjusted life year (DALY), which indicates the loss in health status due to a disease, depression ranked 13th in 2019, the highest in mental disorders (GBD 2019 Mental Disorders Collaborators, 2022). Furthermore, in years of living with disability (YLD), which indicates the number of years living with a disability, it ranked second among all disorders (GBD 2019 Mental Disorders Collaborators, 2022), indicating that it is a chronic condition. As depression is associated with high morbidity and chronicity, it is desirable to devise effective prevention and treatment methods.

Cognitive behavioral therapy (CBT) is an effective psychotherapy for the prevention and treatment of depression. There are various CBTs for depression, but one of the most widely used is that based on the cognitive theory developed by Beck and Rush that assumes cognition is related to the maintenance or exacerbation of depressed mood (Beck et al., 1979). It proposes three types of cognition: dysfunctional attitudes or schemas—cognitive structures formed by childhood experiences; cognitive distortion—a bias in the negative direction of information processing; and automatic thoughts that arise spontaneously in a given situation. Of these, dysfunctional attitudes and automatic thoughts represent thought content, whereas cognitive distortion represents information processing from dysfunctional attitudes to automatic thoughts. A relationship is specified between these three types of thinking (Alloy, 1988). For example, if one has a dysfunctional attitude of not accepting and encounters a situation that is unacceptable to others, the dysfunctional attitude is activated, and cognitive distortions distort information processing to focus on things that are inappropriate to or negative about the situation, resulting in negative automatic thoughts. Cognitive theory suggests that depressive moods are generated or exacerbated by this train of thought.

The Dysfunctional Attitude Scale (Weissman and Beck, 1978) and the Automatic Thoughts Questionnaire (Hollon and Kendall, 1980) were developed to measure dysfunctional attitudes and automatic thoughts, respectively. Japanese versions of these scales have been developed (Sakamoto et al., 2004). Six types of cognitive distortions were identified by Beck et al. (1979) and later expanded to 10 by Burns (1980). Krantz and Hammen (1979) developed a cognitive bias questionnaire (CBQ) for cognitive distortions. The CBQ can assess the degree to which respondents’ thoughts are negatively biased, but it cannot rate which cognitive distortions are strongly experienced by respondents. Therefore, Covin et al. (2011) created the Cognitive Distortion Scale (CDS), which measures the frequency with which respondents experience the 10 types of cognitive distortions. The CDS has good reliability as well as convergent and discriminant validity (Covin et al., 2011; Özdel et al., 2014). It has been translated into other languages (Besta et al., 2014) and is widely used to measure cognitive distortion.

Cognitive theory holds that dysfunctional attitudes increase vulnerability to depression and cause cognitive distortions (Beck et al., 1979). This assumption is supported by the fact that cognitive distortions mediate the relationship between dysfunctional attitudes and depression severity. However, cognitive distortions increase depression severity independent of dysfunctional attitudes (Tecuta et al., 2019). Furthermore, other studies have shown that cognitive distortions increase not only depressive symptoms, but also anxiety symptoms and stress (Buğa and Kaya, 2022).

Clinical benefit of measuring cognitive distortions is recognized because cognitive distortions predict depression severity independent of dysfunctional attitudes. However, cognitive distortions occur unintentionally during the information processing process, self-report measures are questionable. Nonetheless, a meta-analysis examining the association between self-reported measures of cognitive distortion and depression severity found a strong association, indicating the utility of using self-reported measures of cognitive distortion (Nieto et al., 2020).

The clinical benefits of measuring cognitive distortions using a self-reported format have been acknowledged. However, there is no scale in Japan to measure the 10 types of cognitive distortions. In clinical practice, prior identification of the cognitive distortions will help clients recognize distortions in their information processing that they may be unaware of and will allow for a smooth transition to subsequent cognitive interventions. Although research has shown the effectiveness of the treatments targeting cognitive distortion (Jelinek et al., 2016), those on the mechanisms of the treatments has not been promoted because of the lack of scales in Japan. Therefore, this study developed the Japanese version of the CDS (CDS-J) and examined its factor structure, validity, and reliability.

## 2. Materials and methods

### 2.1. Participants

The participants were 271 survey monitors, aged 16–59 years, from an Internet research firm. According to the Consensus-based Standards for the Selection of Health Measurement Instruments (COSMIN), which checks the quality of patient-reported outcomes, the ratio of the number of items to the sample size should be 1:4 to 1:10 when using factor analysis to check the factor structure (Mokkink et al., 2012). Therefore, to confirm the factor structure of the 20-item CDS-J, a sample size of at least 200 individuals was required. In addition, the ratios of the number of participants aged 15–29, 30–44, and 45–59 years were set to 1:1.2:1.6, based on the population ratio in Japan (Statistics Bureau, 2023).

Forty patients with major depression attending a medical facility were included to test discriminant validity. The inclusion was based on the fulfillment of the diagnostic criteria for major depression according to the Diagnostic and Statistical Manual of Mental Disorders, Fifth Edition (DSM-5) (American Psychiatric Association, 2013). The exclusion criterion was presence of psychiatric disorders other than major depressive disorder. Diagnosis was made by a psychiatrist based on the DSM-5 diagnostic criteria.

### 2.2. Measures

#### 2.2.1. Demographics

Participants were asked about their age, sex, and occupation. For occupation, we presented the following options: student, full-time employee, part-time employee, and unemployed, and asked the respondents to check the appropriate box. In addition, participants were asked whether they visited the hospital because of mental problems to exclude any influence of mental problems on the results.

#### 2.2.2. Depression

The Patient Health Questionnaire-9 (PHQ-9) was used to assess depressive symptoms (Kroenke et al., 2001; Muramatsu et al., 2018). The PHQ-9 consists of nine items based on the diagnostic criteria for major depressive disorder in the Diagnostic and Statistical Manual of Mental Disorders, fourth edition (DSM-IV) published by the American Psychiatric Association. A higher total score indicates a higher severity of depression. Severity was rated according to the score as follows: 5–9 = mild, 10–14 = moderate, 15–19 = moderate to severe, and 20–27 = severe (Muramatsu, 2014). The PHQ-9 was used to verify convergent validity.

#### 2.2.3. Anxiety

The Generalized Anxiety Disorder-7 (GAD-7) scale was used to assess anxiety symptoms (Muramatsu, 2014). A higher total score indicate a higher severity of generalized anxiety disorder. Severity was rated as follows: 5–9 = mild, 10–14 = moderate, and 15–21 = severe (Muramatsu, 2014). GAD-7 was used to verify the convergent validity.

#### 2.2.4. Automatic thoughts

The Automatic Thoughts Questionnaire-Revised (ATQ-R) was used to measure automatic thoughts (Kendall et al., 1989; Sakamoto et al., 2004). The ATQ-R measures negative and positive automatic thoughts according to thought content. Higher scores indicate a higher frequency of negative and positive automatic thoughts in the past week. Negative and positive automatic thoughts were used to test convergent and discriminant validities, respectively.

#### 2.2.5. Dysfunctional attitudes

The Dysfunctional Attitude Scale-24 (DAS-24) was used to measure the schema (Tajima et al., 2007), which consists of achievement motivation, other-dependence, and self-control factors. The total scores were used in this study. Higher scores indicate a stronger schema. Dysfunctional attitudes were used to test the discriminant validity.

#### 2.2.6. Cognitive distortions

The CDS was used to measure cognitive distortion (Covin et al., 2011). The CDS measures 10 cognitive distortions: mind reading, catastrophizing, all-or-nothing thinking, emotional reasoning, labeling, mental filter, overgeneralization, personalization, should statements, and minimizing the positive. This scale measures the degree to which these 10 cognitive distortions are likely to occur in social and achievement situations. Higher scores indicate a more frequent occurrence of cognitive distortions.

The CDS-J was developed based on the International Society for Pharmacoeconomics and Outcomes Research (ISPOR) and COSMIN quality checks for patient-reported outcomes (Wild et al., 2005; Mokkink et al., 2012).

First, two authors (TT and MN) independently performed forward translation from English to Japanese. The two versions of the CDS-J were reconciled by TT, MN, and KF, and only minor discrepancies were observed. Discrepancies were discussed until a consensus was reached. A professional English translator, who was an English-Japanese bilingual and blind to the original CDS, translated the provisional CDS-J back into English. The original author evaluated the final English version of the CDS-J and confirmed that the original meaning of each item, instructions, and response were maintained throughout the translation. Finally, seven graduate students whose native language was Japanese and who specialized in psychology were asked to perform a cognitive debriefing of the CDS-J to make minor corrections to word endings. Thus, the CDS-J was prepared.

### 2.3. Procedure

To confirm the reliability and validity, the CDS-J, PHQ-9, GAD-7, ATQ-R, and DAS-24 were administered to 271 registered monitors of an Internet research company. In addition, the CDS-J was administered again to 71 participants of the first survey after 2 weeks to examine retest reliability. The CDS-J, PHQ-9, and GAD-7 were administered to 40 patients with major depression to verify the discriminant validity.

### 2.4. Statistical analysis

The data for 237 of 271 participants were included in the analyses after removing the data for 34 participants currently attending medical institutions.

To confirm the factor structure of the CDS-J, a one-factor model based on the results of previous studies (Covin et al., 2011; Besta et al., 2014; Özdel et al., 2014) and a 10-factor model based on the results of Martskvishvili et al. (2021), and a confirmatory factor analysis were performed using the MLR estimator. Four fit indices were employed: chi-square (*χ*^2^), comparative fit index (CFI), root mean square error of approximation (RMSEA), and standardized root mean square residual (SRMR). The goodness of fit criteria were set as CFI > 0.90, RMSEA <0.10, and SRMR <0.10 (Bentler and Bonnet, 1980; Browne and Cudeck, 1992; Kline, 2005).

To examine normality of the total score of CDS-J, PHQ-9, GAD-7, DAS-24, negative automatic thoughts, and positive automatic thoughts, we carried out a Kolmogorov–Smirnov test. In addition, to examine outlier, we carried out a Grubbs test.

To examine reliability, the *ω* for the CDS-J was calculated. To examine the retest reliability, Spearman’s rank correlation coefficient was calculated for the 71 participants for whom data were collected 2 weeks after the initial implementation of the CDS-J.

To test convergent validity, Spearman’s rank correlation coefficients were calculated for the CDS-J and the total scores of the PHQ-9, GAD-7, negative automatic thoughts on the ATQ-R, and DAS-24. To verify discriminant validity, in addition to calculating Spearman’s rank correlation coefficients between the CDS-J and positive automatic thoughts on the ATQ-R, the Mann–Whitney U test was conducted on the total scores of the CDS-J between participants who were healthy and those with major depression. The effect sizes was defined as follows: 0.20–0.50 = small, 0.50–0.80 = medium, and ≥ 0.80 = large. SPSS version 29 (IBM Corp., 2022), R 4.2.2 package lavaan 0.6-14 and semplots were used for the analyses (Jorgensen et al., 2022; R core team, 2022; Epskamp, 2022).

### 2.5. Ethical consideration

Participants were considered to have provided written informed consent for the use of the information provided for research purposes by completing the research questionnaire. This study was approved by the Ethical Review Committee of Psychological Sciences at the University of Human Environments.

## 3. Results

### 3.1. Demographic data

Demographic data of the participants are presented in Table 1. The number of participants by age group was consistent with the population ratio in Japan. Among the participants, 110 (46%) had mild or higher severity depressive symptoms and 82 (35%) had mild or higher severity anxiety symptoms.

**Table 1:** Demographic charactaristics.

*N* (male/female)	237 (121/116)
Age	39.84 ± 12.20
15–29	68
30–44	74
45–59	95
Work	
Full-time	127
Part-time	52
Student	22
Unemployed	36
Depression	
Mild	61
Moderate	28
Moderate–severe	11
Severe	10
Anxiety	
Mild	48
Moderate	19
Severe	15

± 1 standard deviation.

### 3.2. Descriptive statistics and internal consistency

Descriptive statistics and internal consistency are shown in Table 2. Normality was not recognized except for DAS-24. Therefore, the subsequent tests used a method that did not assume normality. As a result of the Grubbs test, no outlier was observed in the total score of the CDS-J. The ω for the total CDS-J score was 0.96, indicating high internal consistency. The ω of the scales used for convergent and discriminant validity were high, ranging from 0.91–0.98.

**Table 2:** Descriptive statistics.

		Healthy			Depression		
		Mean	SD^f^	ω^g^	Mean	SD^f^	ω^g^
CDS-J^a^	Total	60.47	24.84	0.96	88.97	30.13	0.96
	Mind reading	6.55	2.99	0.87	9.85	3.65	
	Catastrophizing	5.89	3.13	0.90	8.69	3.66	
	All-or-nothing thinking	5.66	3.04	0.91	7.74	3.68	
	Emotional reasoning	6.25	2.92	0.88	9.36	3.51	
	Labeling	6.01	3.01	0.90	8.92	4.04	
	Mental filter	5.95	3.10	0.91	8.82	4.00	
	Overgeneralization	6.00	3.11	0.92	8.59	3.82	
	Personalization	6.08	3.10	0.88	9.08	3.69	
	Should statement	6.14	3.06	0.89	9.85	3.52	
	Minimizing the positive	5.94	3.02	0.92	8.08	3.58	
DAS-24^b^	Total	82.04	24.25	0.91			
ATQ-R^c^	Negative automatic thought	56.56	28.18	0.98			
	Positive automatic thought	20.27	8.41	0.91			
PHQ-9^d^	Total	5.67	6.11	0.91	13.23	7.52	
GAD-7^e^	Total	4.16	5.11	0.94	9.38	5.96	

a Japanese version of the cognitive distortion scale.

b Dysfunctional attitude scale-24.

c Automatic thoughts questionnaire-revised.

d Patient health questionnaire-9.

e Generalized anxiety disorder-7.

f Standard deviation.

g Omega.

### 3.3. Confirmation of factor analysis

To confirm the factor structure of the CDS-J, we performed confirmatory factor analysis using the MLR estimator. First, a one-factor model was examined. The results showed that the model fit poorly with *χ*^2^ (170) = 873.24, *p* < 0.01, CFI = 0.73, RMSEA = 0.18, and SRMR = 0.07.

Next, a 10-factor model was examined. The results showed a better model fit with χ^2^(125) = 397.88, *p* < 0.01, CFI = 0.91, RMSEA = 0.12, and SRMR = 0.03.

**Figure 1:**
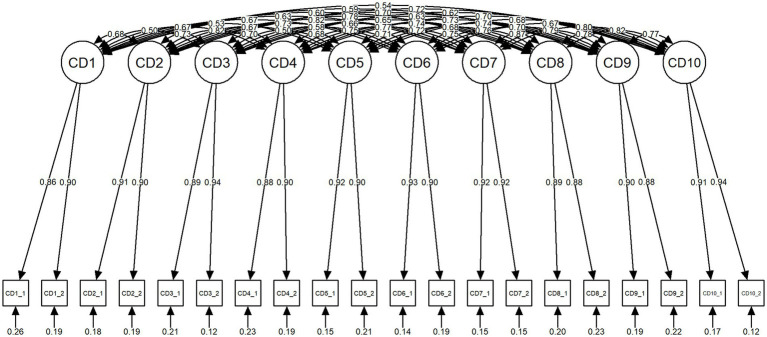
Final model of the CDS-J. CD, cognitive distortion; CD1, mindreading; CD2, catastrophizing; CD3, all-or-nothing thinking; CD4, emotional reasoning; CD5, labeling; CD6, mental filter; CD7, overgeneralization; CD8, personalization; CD9, should statement; CD10, minimizing the positive; CD_1, interpersonal situation; CD_2, achievement situation; CDS-J, Cognitive Distortion Scale-Japanese version.

Additionally, the 10-factor model exhibited inadequate RMSEA values. However, it was adopted because of its better fit than that of the one-factor model and its consistency with cognitive theory (Figure 1).

### 3.4. Convergent and discriminant validity

To test convergent validity, Spearman’s rank correlation analysis was conducted on the total scores of the CDS-J, DAS-24, negative automatic thoughts on the ATQ-R, PHQ-9, and GAD-7. The results showed that the CDS-J had a significant positive correlation with all scales (all *p* < 0.01, Table 3).

**Table 3:** Convergent and discriminant validity of CDS-J.

	CDS-J	DAS-24	NAT	PAT	PHQ-9	GAD-7
CDS-J^a^	1.00					
DAS-24^b^	0.39^**^	1.00				
NAT^c^	0.45^**^	0.45^**^	1.00			
PAT^d^	−0.07	−0.14^*^	−0.14^*^	1.00		
PHQ-9^e^	0.44^**^	0.41^**^	0.68^**^	−0.34^**^	1.00	
GAD-7^f^	0.43^**^	0.49^**^	0.68^**^	−0.25^**^	0.77^**^	1.00

** *p* < 0.01, * *p* < 0.05.

a The Japanese version of the cognitive distortion scale.

b Dysfunctional attitude scale-24.

c Negative automatic thought.

d Positive automatic thought.

e Patient health questionnaire-9.

f Generalized anxiety disorder-7.

To test discriminant validity, Spearman’s rank correlation analysis was conducted with the total CDS-J score and positive automatic thoughts on the ATQ-R. There was no significant correlation between the two variables (*p* = 0.26).

In addition, data were obtained from 40 patients with major depression to determine whether differences in the CDS-J scores were observed between participants who were healthy and those with major depression; however, one patient provided an incomplete response. Therefore, data for 39 patients (12 men and 27 women, mean age 39.69 ± 12.52 years) were included in the analysis.

Descriptive data for participants with major depression are presented in Table 2. The total CDS-J score, and all factor scores of healthy participants and those with major depression were compared. The results showed significant differences in all scores (all *p* < 0.05). These results indicated that the CDS-J had adequate convergent and discriminant validity.

### 3.5. Retest reliability

The CDS-J was administered to 71 participants again after 2 weeks. The correlation coefficient between the first and second CDS-J total scores was significant (*p* = 0.61, *r* < 0.01), indicating a moderate strength of association.

## 4. Discussion

This study aimed to develop the CDS-J and to test its factor structure, reliability, and convergent and discriminant validity. The results of the confirmatory factor analysis generally supported a 10-factor structure. The CDS-J has a high internal consistency. Furthermore, it showed moderately significant associations with dysfunctional attitudes, negative automatic thoughts, and severity of depressive and anxiety symptoms, indicating convergent validity. In contrast, it had no significant association with positive automatic thoughts, was significantly different between participants without and with major depression, and had discriminant validity. The scale has a certain level of reliability. These results suggest that the CDS-J has adequate reliability and validity.

Covin et al. (2011) exploratively confirmed the one-factor structure of CDS in college students. Furthermore, Özdel et al. (2014) exploratively confirmed the one-factor structure in participants without and those with major depression. However, Martskvishvili et al. (2021) compared the one-factor model presented in previous studies with a 10-factor model fitted to a theoretical model and adopted the 10-factor model. Similarly, the results of this study clarified that the CDS-J has a 10-factor structure. These results indicate that the CDS-J can measure 10 types of cognitive distortions.

The *ω* coefficient for the total CDS-J score was 0.96, indicating higher internal consistency than that in studies by Covin et al. (2011) and Özdel et al. (2014). The retest reliability of the CDS-J was examined using Spearman’s rank correlation analysis and a moderately significant positive correlation was observed. These results indicated that the CDS-J had the same or even higher internal consistency than the original CDS. Furthermore, the retest reliability results for the CDS-J suggested that cognitive distortions are stable cognitive variables to some extent. Conventional cognitive theory considers dysfunctional attitudes to be trait-based and automatic thoughts to be state-dependent, and does not specifically address cognitive distortions. According to conventional theoretical models, cognitive distortions occur when dysfunctional attitudes are activated and can be considered state-dependent. In contrast, in schizophrenia, another psychiatric disorder, delusion severity is associated with a type of cognitive distortion known as jumping to conclusions bias (JTC) (Ross et al., 2015). Furthermore, JTC occurs not only in the acute phase of active delusions but also in the information processing of patients who are not currently delusional (Moritz and Woodward, 2005), suggesting that it may be present as a trait. Given the results of studies on other psychiatric disorders, it is conceivable that cognitive distortions associated with symptoms may be characteristics of information processing, suggesting that they may also be characteristics of cognitive distortions related to the severity of depression.

The CDS-J showed high convergent validity as in the original version. According to cognitive theory, the activation of dysfunctional attitudes produces cognitive distortions and negative automatic thoughts (Beck et al., 1979). Cognitive distortion exacerbates depressive symptoms (Tecuta et al., 2019). In other words, cognitive distortion is a cognitive concept associated with dysfunctional attitudes, negative automatic thoughts, and depressive symptoms; which was found in the present study.

The CDS-J showed high discriminant validity similar to the original version. The original version showed no significant correlation with positive automatic thoughts (Covin et al., 2011), and differences in total scores were found between participants with and without major depression (Özdel et al., 2014). These findings were consistent with those of the present study on CDS-J.

The results of this study indicate that the CDS-J has sufficient reliability and validity, and can be used clinically and in research studies targeting cognitive distortions in major depression.

### 4.1. Limitation

This study had several limitations. First, an online survey was conducted; there, measurement accuracy may be an issue (Miura and Kobayashi, 2018). Miura and Kobayashi (2018) highlighted that internet surveys are likely to generate guesswork to minimize the effort on responses. In this study, we addressed this problem by a question that asked respondents to always answer 1 and only included those who responded as instructed; however, there may have been a small effect of conducting the survey online. Second, the patients with major depression in this study were identified with a single diagnosis according to the DSM-5. However, structured interviews were not conducted according to the diagnostic criteria. The diagnostic concordance rate for psychiatric diagnoses is not always high (Freedman et al., 2013). This raises questions regarding the diagnosis of patients with major depression. In future, when testing the structure and reliability of the CDS-J in patients with major depression, patients should be selected through structured interviews. Third, the χ2 test and RMSEA did not meet the criteria. The χ2 test is sensitive to the sample size, even with a that of a few hundreds. Slight deviations between the model and data can cause rejection of the model (Murakami and Yukihito, 2018). The sample size for this study was 237; therefore, it may have affected the analyses. The RMSEA was 0.12, slightly above the criterion of 0.10. Although it is difficult to interpret RMSEA, it is possible that the 10-factor model of cognitive distortion derived from cognitive theory and factor analysis may differ from the measured values. The structure of cognitive distortion requires further investigation.

### 4.2. Conclusion

A Japanese version of the CDS was constructed and its reliability and validity were confirmed in this study. The CDS-J was confirmed to have a 10-factor structure. Moreover, its convergent and discriminant validity was demonstrated, and the scale showed high test–retest reliability. The CDS-J covers multiple types of cognitive distortions, such as mind reading, catastrophizing, all-or-nothing thinking, emotional reasoning, labeling, mental filter, overgeneralization, personalization, should statements, and minimizing the positive; this is an advantage of the CDS-J. The use of the CDS-J is expected to promote further research on cognitive distortion in the treatment and prevention of depression.

## Data availability statement

The raw data supporting the conclusions of this article will be made available by the authors, without undue reservation.

## Ethics statement

The studies involving humans were approved by the Ethical Review Committee of Psychological Sciences at the University of Human Environments. The studies were conducted in accordance with the local legislation and institutional requirements. Written informed consent for the inclusion of healthy participants was not required from the participants and/or participants’ legal guardians/next of kin in accordance with the local legislation and institutional requirements. Written informed consent for the inclusion of the clinical sample was provided by the participants.

## Author contributions

TT: Conceptualization, Data curation, Formal analysis, Funding acquisition, Investigation, Methodology, Project administration, Resources, Writing – original draft, Writing – review & editing. KF: Conceptualization, Data curation, Formal analysis, Investigation, Methodology, Writing – review & editing. MN: Conceptualization, Formal analysis, Investigation, Methodology, Writing – review & editing. HU: Conceptualization, Investigation, Methodology, Project administration, Supervision, Writing – review & editing. KN: Conceptualization, Data curation, Investigation, Methodology, Project administration, Supervision, Writing – review & editing.
